# Non-destructive monitoring of root biomass in hydroponically grown leafy vegetables: comparison between machine learning-based RGB and hyperspectral imaging

**DOI:** 10.1186/s13007-026-01515-8

**Published:** 2026-03-10

**Authors:** Ziyi Jin, Daisuke Yasutake, Tadashige Iwao, Yuki Sago, Gaku Yokoyama, Shigehiro Kubota, Tomoyoshi Hirota

**Affiliations:** 1 Graduate School of Bioresource and Bioenvironmental Sciences, Kyushu University, Fukuoka, 819-0395 Japan; 2 Faculty of Agriculture, Kyushu University, Fukuoka, 819-0395 Japan; 3 IoP Co-Creation Center, Kochi University, Nankoku, 783-8502 Japan; 4 Faculty of Agriculture, Yamaguchi University, Yamaguchi, 753-8515 Japan

**Keywords:** Partial least squares regression, Convolutional neural network, Root dry weight, Hydroponic cultivation, Phenotyping, Spinach

## Abstract

**Background:**

Root biomass serves as a critical indicator of plant eco-physiological status and crop productivity, yet its non-destructive monitoring remains challenging because of its underground location. The use of transparent nutrient film technique (NFT) systems enables direct observation of entire root systems, rendering image-based phenotyping feasible. In this study, we investigated and compared the performance of RGB and hyperspectral imaging for predicting root dry weight in hydroponically grown spinach (*Spinacia oleracea* L.).

**Results:**

Using 430 root segments divided from 60 plants, three models were developed: (1) an area-based regression based on root coverage, (2) a convolutional neural network (CNN) using RGB images, and (3) a partial least squares regression (PLSR) model using hyperspectral data (450–950 nm). The area-based regression exhibited limited accuracy (*R*² = 0.446) because of saturation at high root coverage. The CNN model improved predictive performance (*R*² = 0.739) but tended to overestimate sparse roots as a result of resolution constraints. The PLSR model achieved the highest accuracy (*R*² = 0.822, RMSE = 0.019 g/segment), with significantly lower error than RGB-based approaches (*P* < 0.01). Variable importance in projection analysis indicated that PLSR effectively exploited spectral signatures at 450 nm (background contrast) and 750 nm (tissue scattering), thereby maintaining stable accuracy across the full biomass range. When validated using 104 independent plants, the PLSR model achieved high predictive accuracy. Furthermore, as a proof of concept, this model successfully visualized the spatiotemporal dynamics of root biomass accumulation over 50 days, with only a 7.70% relative error at harvest.

**Conclusions:**

To our knowledge, this study is among the first to demonstrate the non-destructive monitoring of biomass distribution within entire root systems under production conditions. Hyperspectral imaging combined with PLSR outperforms RGB-based approaches by capturing spectral signatures that reflect internal tissue properties of roots, thereby overcoming limitations caused by morphological occlusion. This approach provides a robust tool for precision agriculture and high-throughput phenotyping, enabling continuous assessment of root growth through simple modifications to the existing hydroponic systems.

**Graphical Abstract:**

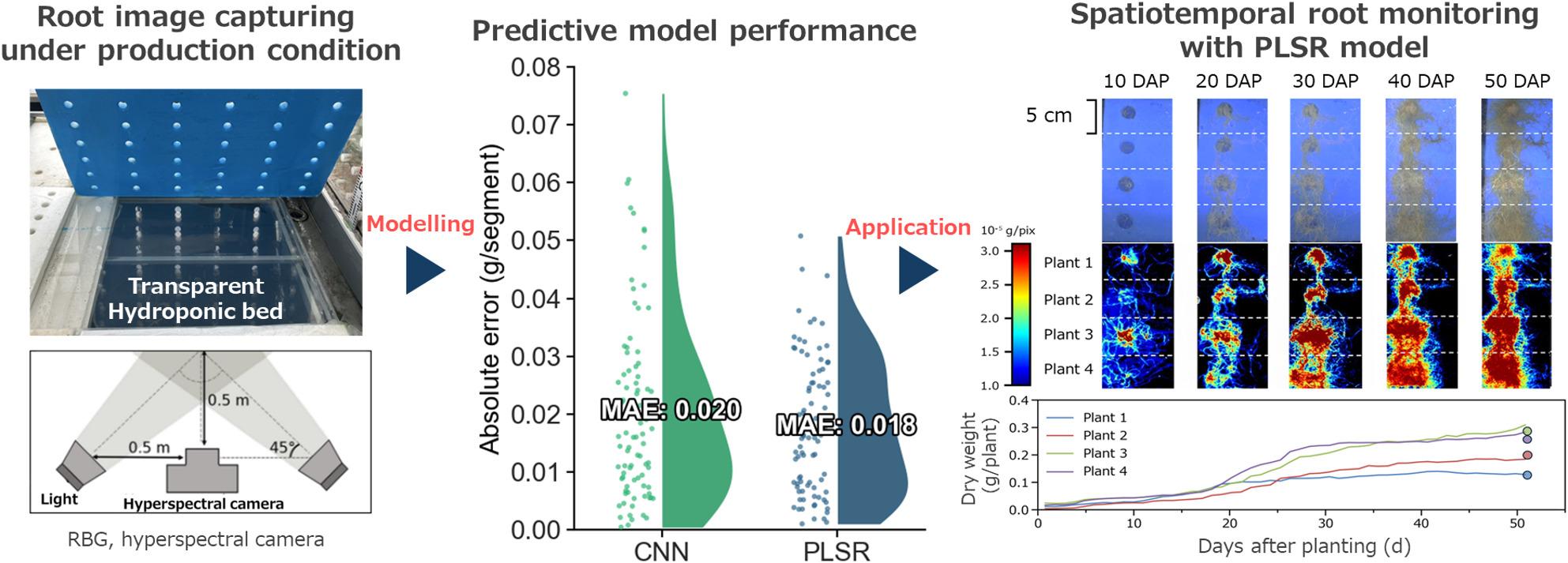

**Supplementary Information:**

The online version contains supplementary material available at 10.1186/s13007-026-01515-8.

## Background

Roots are fundamental organs that support plant growth by absorbing and transporting water and essential nutrients from the cultivation medium. They act as sinks for photosynthetic products, contributing 20%–80% of total plant dry weight [1, 2], and they can also remobilize the stored products to the shoot, thereby serving as a source [3]. Thus, root biomass characteristics, such as dry weight and the distribution architecture of root systems, are indispensable indicators of plant eco-physiological traits, as they reflect patterns of resource acquisition and allocation. Moreover, depending on crop type, root biomass is more sensitive to rhizosphere environmental stresses, such as excess nutrients and/or minerals [4, 5], oxygen deficiency [6], and high and/or low pH [7], relative to above-ground biomass. Root biomass is also correlated with crop yield [8], and considering root biomass together with shoot biomass can improve the accuracy of yield prediction [9]. Consequently, monitoring root biomass contributes to a more comprehensive understanding of plant vitality and is effective for data-driven crop production, such as smart and precision agriculture.

Despite its importance, root biomass remains difficult to monitor because it is located underground. Although destructive methods, such as collecting and weighing root samples, provide direct and reliable measurements, they are labor-intensive and generate only discrete data, which limits their applicability for continuous monitoring. To address this limitation, roots have been observed through transparent interfaces inserted vertically into cultivation media (above-ground rhizotron, minirhizotron, rhizobox, etc.), enabling non-destructive monitoring of root biomass using image analysis [10, 11]. However, despite allowing non-destructive observation, the opacity of cultivation media restricts measurements to only part of the root system, which may not accurately represent total root biomass, because root system architecture is heterogeneous and composed of individual roots with different diameters and functions. Other non-destructive approaches, such as X-ray and three-dimensional scanning, have also been applied to monitor root biomass, but these techniques typically require either small-scale pot cultivation or large-scale measurement systems [12, 13], making them difficult to apply under actual production conditions. To address these limitations, hydroponic cultivation systems might offer a promising alternative to overcome these constraints. By modifying the nutrient film technique (NFT) bed, which is commonly used in crop production, with a transparent acrylic baseplate, entire root systems can be observed [14]. Although spatial distribution and growth patterns of roots differ between soil-based and hydroponic production systems, the fundamental challenge of root invisibility remains universal across cultivation methods. This methodological framework may provide insights to advance root phenotyping in diverse production systems through image-based monitoring approaches.

In recent years, image-based techniques, particularly RGB and hyperspectral imaging, have attracted increasing attention for plant biomass prediction. Declining camera costs, advances in mathematical analysis methods, and improvements in computational power have further accelerated progress in image-based analysis. Consequently, image-based approaches now span a wide range of methodologies, from fundamental image processing to advanced machine learning techniques [15].

Among these image-based approaches, RGB imaging has been widely adopted as a cost-effective method and has been successfully applied to estimate above-ground biomass. For instance, early studies employed image segmentation to separate plant features from the background and then used the derived plant coverage to construct regression models for biomass prediction [16, 17]. With continued advances in computational power, deep learning techniques, such as convolutional neural networks (CNNs), have been introduced into this field [18]. These models can automatically learn and extract complex morphological and textural features from RGB images, thereby enabling more accurate prediction of above-ground dry weight of crops [19, 20]. However, RGB-based approaches often show reduced predictive accuracy when substantial leaf overlap occurs [21]. Beyond above-ground applications, several studies have reported significant correlations between RGB root images and root dry weight [10, 11]. Nevertheless, these studies largely remained at the level of correlation analysis and did not develop predictive models for root dry weight.

Relative to RGB imaging, which records reflectance information in only three broad bands corresponding to the red, green, and blue regions, hyperspectral imaging (HSI) acquires reflectance information across hundreds of contiguous, narrow spectral bands—for example, from the visible to near-infrared range. This information results in high-dimensional datasets containing detailed spectral signatures of plants and enables the detection of signals associated not only with morphological features but also with key biochemical parameters. For instance, HSI has been used to estimate leaf water content, providing valuable indicators of drought stress [22, 23]. In addition, HSI has been successfully applied to quantify nitrogen content, thereby offering a non-destructive approach for analyzing nutrient use efficiency [24, 25]. To effectively extract quantitative information from such high-dimensional hyperspectral data, appropriate mathematical methods including machine-learning are required. Among these methods, partial least squares regression (PLSR) has become one of the most widely used techniques [26]. PLSR is particularly well-suited to hyperspectral applications because it reduces high-dimensional variables to a limited number of latent variables while minimizing multicollinearity, and it has demonstrated strong performance in predicting above-ground plant dry weight using spectral data [27, 28]. However, no studies have specifically examined the relationship between hyperspectral images and root dry weight. If an appropriate approach is available to observe entire root systems, HSI may also provide insights into root dry weight and its distribution architecture within root systems, analogous to its established applications for above-ground traits.

Given the unique observation environment provided by NFT systems modified with a transparent baseplate, together with the capabilities of HSI, we hypothesized that machine learning-based RGB imaging and hyperspectral imaging could predict root dry weight non-destructively under actual production conditions, with HSI expected to achieve higher accuracy because of its richer spectral information. Leafy vegetables were selected as the target crop since their growth is predominantly vegetative, without complex reproductive phases, making them suitable for this initial stage of method validation. To test this hypothesis, we developed predictive models of root dry weight using RGB and hyperspectral imaging data. Subsequently, we compared the predictive accuracy of different modeling approaches, including a simple regression model based on the ratio of root area in the image, a CNN model, and a PLSR model. We subsequently applied the model with the highest predictive accuracy to visualize root dry weight throughout the entire growth period, thereby demonstrating the system’s potential for continuous monitoring of root biomass dynamics as a proof of concept. To the best of our knowledge, this work demonstrates one of the first non-destructive monitoring of biomass distribution within the entire root system under production conditions, extending image-based approaches previously applied to above-ground biomass, and contributes to data-driven, efficient crop production. In addition, beyond agricultural applications, non-destructive monitoring of root biomass has important implications for plant eco-physiological research.

## Materials and methods

### Model development

#### Plant material and growth conditions

**Fig. 1:**
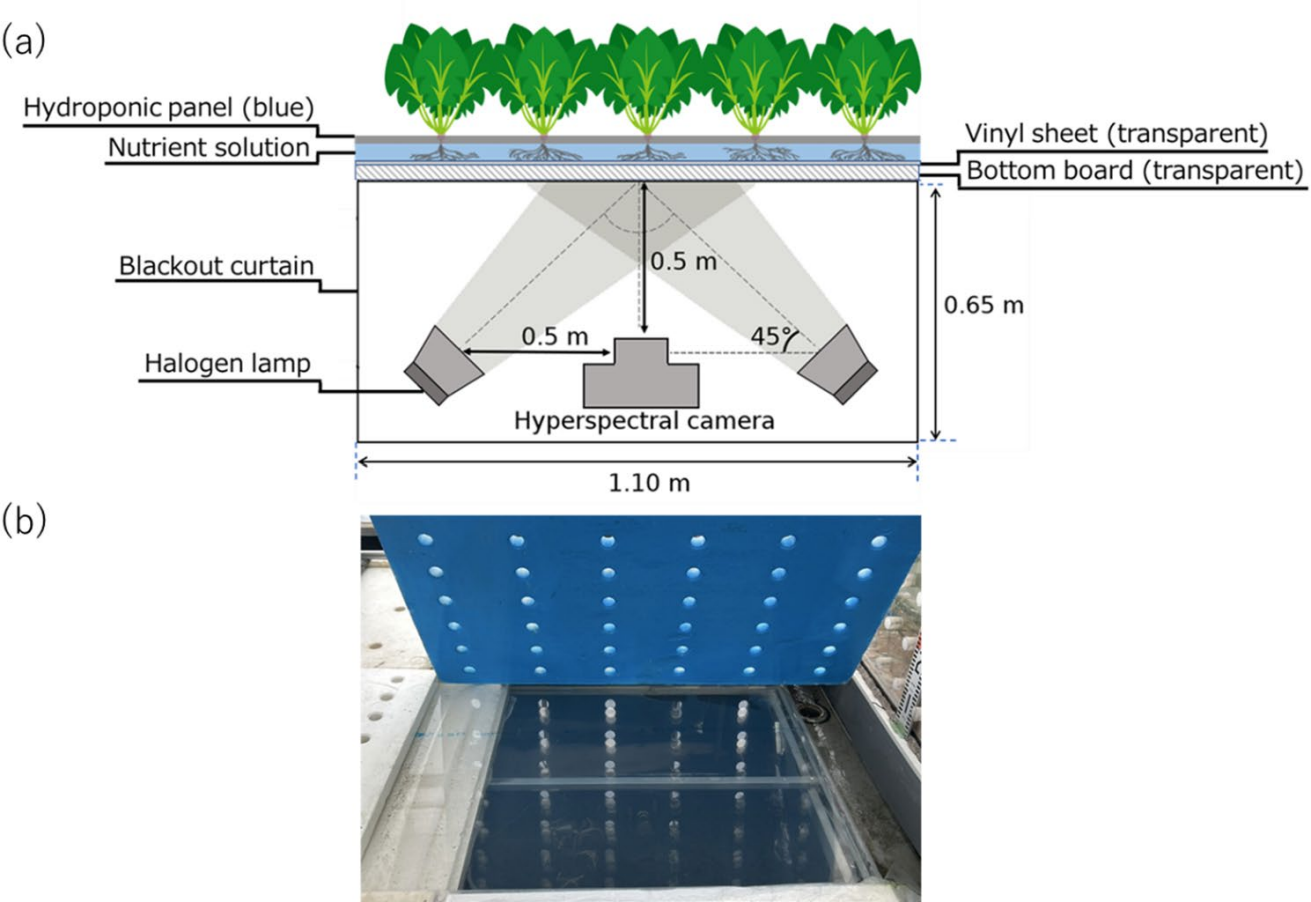
Schematic diagram (**a**) illustrating spectral reflectance measurement of roots using a hyperspectral camera on a section of the NFT hydroponic bed, which consisted of a hydroponic panel with its underside painted blue, a transparent polyolefin sheet, and a transparent acrylic board. Top-down photograph (**b**) of the transparent hydroponic bed with the hydroponic panel lifted. The nutrient solution flowed between the hydroponic panel and the polyolefin sheet. The hyperspectral camera and four halogen lamps were positioned beneath the NFT bed and enclosed with a blackout curtain. NFT, nutrient film technique

Spinach (*Spinacia oleracea* L., cv. ‘Wase Kurone Horenso’), a representative leafy vegetable, was used as plant material and cultivated in an NFT hydroponic system in a high-eaves greenhouse at the Ito Plant Experimental Fields and Facilities, Faculty of Agriculture, Kyushu University, Japan (Lat. 33°59.3′N, Long. 130°21.5′E). The greenhouse was operated under natural light conditions and equipped with environmental control systems, including roof and side-window ventilation that was activated when the air temperature exceeded 22 °C. It was also equipped with a heater (HK2027TEV, NEPON Inc., Tokyo, Japan) that was operational when the temperature fell below 8 °C. To improve the generalizability of the prediction models, model development was based on plant segments collected during two cultivation periods in different seasons, November 5–December 15, 2022 (24 plants) and March 10–April 15, 2024 (36 plants). During the first period, the average air temperature and daily light integral were 15.3 °C and 6.7 mol m⁻² d⁻¹, respectively, whereas during the second period, these values were 18.0 °C and 9.0 mol m⁻² d⁻¹. In both periods, seeds were sown on urethane cubes and raised in the same greenhouse, and seedlings were transplanted into the NFT system 10 days after sowing. A total of 60 plants were grown under consistent root-zone conditions, with a nutrient solution maintained at an electrical conductivity (EC) of 2.0 dS m⁻¹, a pH of 6.0, and a temperature of 20 °C. The solution contained 17 mmol L⁻¹ NO₃⁻, 1.1 mmol L⁻¹ PO₄³⁻, 8.4 mmol L⁻¹ K⁺, 3.9 mmol L⁻¹ Ca²⁺, 1.6 mmol L⁻¹ SO₄²⁻, and 1.5 mmol L⁻¹ Mg²⁺. To facilitate root imaging, the bottom of the NFT bed and the covering film were replaced with transparent materials, allowing the roots to be imaged from underneath (Fig. 1). To facilitate clear discrimination between roots and background in the images, the hydroponic panels were painted blue [29]. This procedure followed the method previously described by Jin et al. [14].

**Fig. 2:**
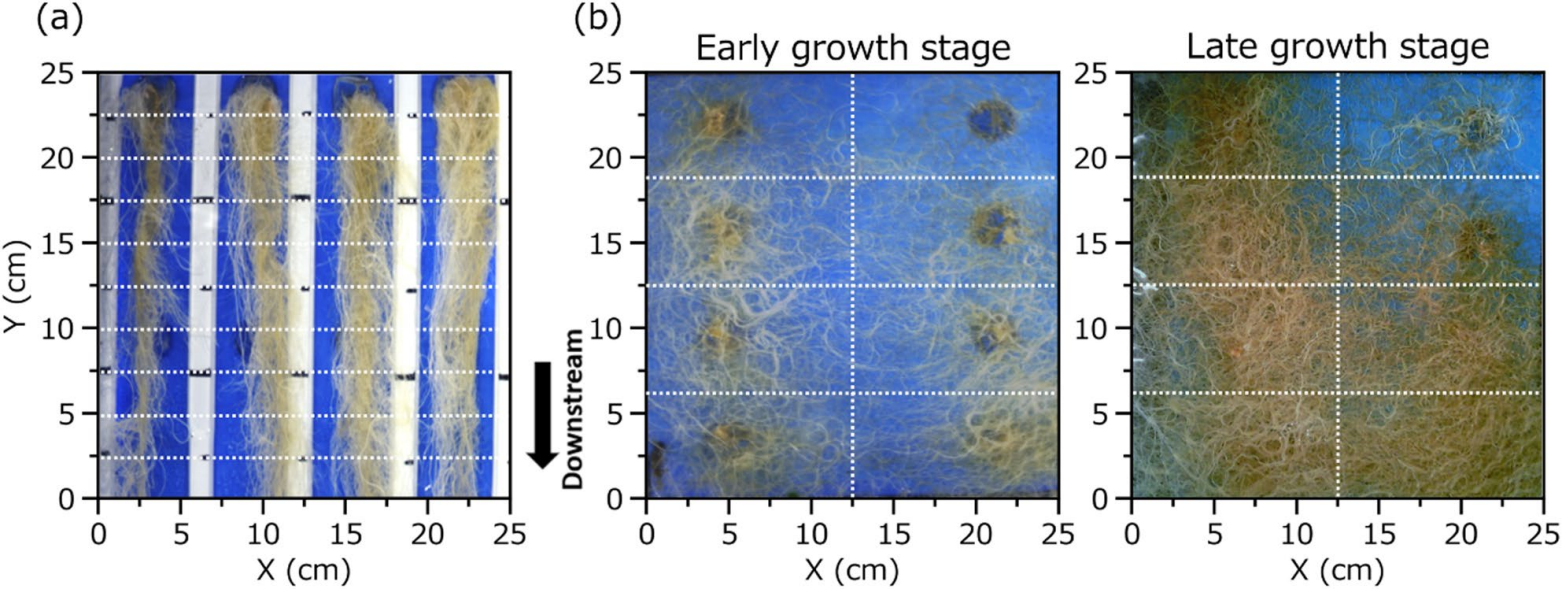
Photographs of plant root growth patterns obtained for segments collection under a linear growth condition used for model development (**a**) and under a natural root development condition used for model applicability (**b**). The white dashed line indicates the cutting line

To preserve root shape during sampling, roots were guided to grow downstream in a linear orientation by installing partition boards between plants (Fig. 2). These boards served two purposes: physically separating root systems to prevent entanglement and providing visual markers at 2.5 cm intervals to enable accurate segmentation and sampling. In addition, the boards provided structural support that stabilized root orientation during cultivation. Without this support, roots frequently shifted or curled when the cultivation panel was lifted for sampling and dry weight measurement, particularly during early growth stages. This instability hindered the preservation of the original root orientation and reduced the consistency of segment-based sampling. Using this setup, each plant’s root system was divided into 7–9 segments, each 5 cm wide and 2.5 cm long, depending on total root length, resulting in a total of 430 root segments. It should be noted that this linear root arrangement may increase spatial overlap of roots relative to conventional radial expansion patterns under natural root development conditions. Here, 20 segments in which roots extended excessively beyond the partition boards were excluded from analysis and most of those segments were near the plant base. This exclusion also contributed in eliminating cases with abnormally high root overlap that would not occur under natural root development conditions. The retained 430 segments encompassed a broad range of root densities, from dense upstream segments to sparse downstream regions, enabling the models to learn across varying levels of overlap.

#### Image acquisition and root sampling

Prior to root sampling, images were acquired from the underside of the NFT bed using a hyperspectral camera (Specim IQ, Specim Ltd., Oulu, Finland) capable of capturing hyperspectral (400–1000 nm, 204 bands) and RGB images simultaneously. The camera was positioned 0.5 m below the bottom board (Fig. 1), resulting in a field of view of 25 × 25 cm (512 × 512 pixels). Imaging was conducted after sunset to avoid interference from ambient light. Four halogen lamps (JDR Φ50, 30 W, Phoenix Electric Co., Ltd., Japan) were activated only during image acquisition to provide consistent illumination, while the underside of the NFT system remained covered with blackout curtains at all times. For radiometric calibration, a standard white reference panel was photographed prior to root data acquisition under identical illumination conditions. Dark current correction was applied automatically by the camera’s internal system to account for sensor noise. By normalizing spectral reflectance relative to the white reference, this calibration procedure mitigates the impact of variation in light source characteristics, thereby ensuring robust measurements under changing lighting conditions. Spectral data in the range of 450–950 nm were selected to reduce noise at the spectral edges. RGB images were acquired simultaneously using the built-in RGB camera sensor integrated into the hyperspectral camera system (Specim IQ). This configuration enabled direct comparison of conventional RGB imaging technology with hyperspectral imaging to assess their respective practical applicability in production environments.

Following image acquisition, root segments were harvested by cutting along the predefined 2.5 cm partition markers. The segments were subsequently oven-dried at 80 °C for 72 h before being weighed. These measurements were used as ground-truth data for model development and validation.

#### Model construction

We constructed three different models to estimate root dry weight using RGB and/or hyperspectral images. For all three models, we used 80% of the segments as a training dataset.

The first model was an area-based regression model using RGB images. Binarization was applied to the RGB images to distinguish roots from the background using Otsu’s thresholding method [30], and the proportion of root pixels within each segment area was calculated and used as the explanatory variable in a simple regression model. Area-based methods have been widely applied in plant phenotyping owing to their simplicity and computational efficiency [16, 17]. This model therefore functions as a straightforward baseline for comparison with more advanced deep learning and spectral analysis approaches.

**Fig. 3:**
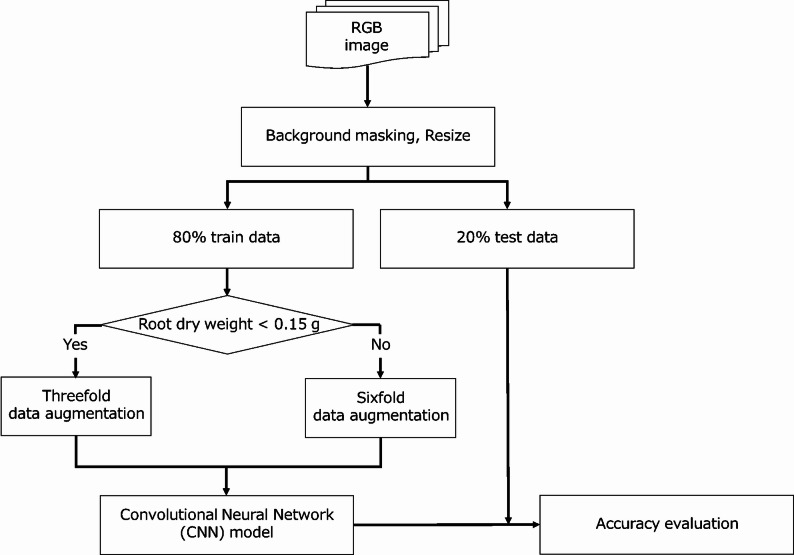
Flowchart summarizing the CNN modeling process using RGB images. *CNN* convolutional neural network

The second model was a CNN model using RGB images (Fig. 3). Background regions were removed from all images prior to model construction to isolate root features. To ensure consistency of model input, raw images were resized to standardized dimensions of 224 × 224 pixels. Pixel values were normalized to the range from 0 to 1 by division by 255. Data augmentation was performed by horizontally and vertically flipping the original RGB images. Root segments (5 × 2.5 cm) with a root dry weight of ≤ 0.150 g/segment were augmented threefold, whereas segments with a dry weight of > 0.150 g/segment were augmented sixfold, resulting in a final training dataset of approximately 1638 augmented images. The CNN model was constructed using MobileNetV2 [31], pre-trained on ImageNet [32]. The convolutional base, up to the final 7 × 7 × 1280 feature map, was retained, and its weights were frozen. The original classifier was replaced with a custom regression head consisting of a Global Average Pooling 2D layer, a dense layer with 128 neurons and ReLU activation, a Dropout layer (rate = 0.3), and a single-neuron output layer with Sigmoid activation to predict normalized root dry weight. The model was trained using TensorFlow 2.8 [33] and the Adam optimizer [34] with default parameters to minimize mean squared error. Training was conducted for 100 epochs with a batch size of 32, and the weights corresponding to the lowest validation loss were selected for testing. To optimize convergence and prevent overfitting, a learning rate scheduler and an early stopping mechanism with a patience of ten epochs were employed during training.

**Fig. 4:**
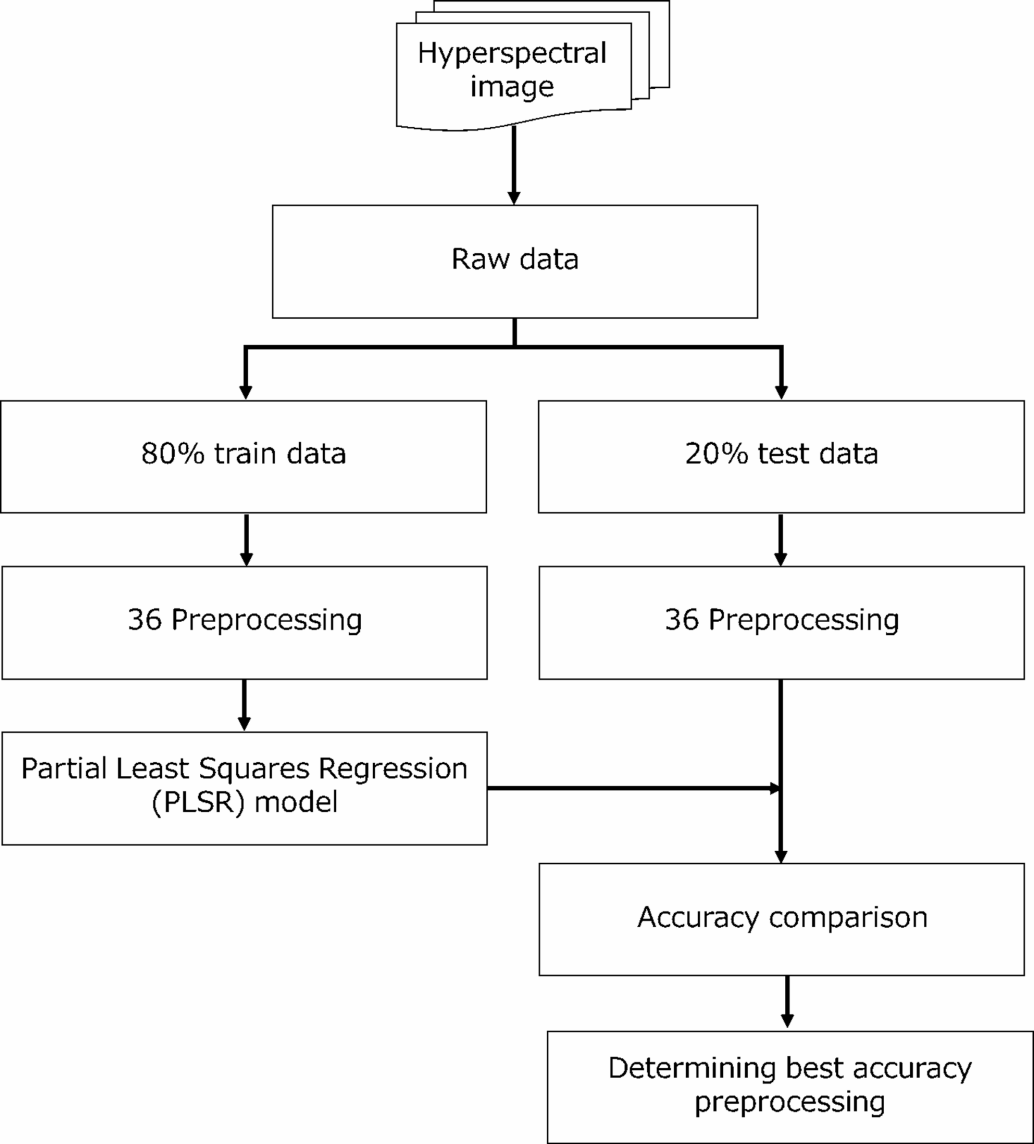
Flowchart summarizing the modeling process using hyperspectral images

The third model was a PLSR model constructed using hyperspectral images (Fig. 4). Reflectance spectra were extracted from these hyperspectral images, and then we applied 36 combinations of preprocessing methods [35–39] to the spectral data (Table 1). For each preprocessing combination, a PLSR model was constructed to estimate root dry weight. Prior to modeling, the target variable, root dry weight, was subjected to a square root transformation to improve data normality. PLSR models were constructed using the OScoresPLS algorithm. To prevent overfitting, the optimal number of latent variables (LVs) was determined based on the minimum root mean squared error of cross-validation obtained from leave-one-out cross-validation on the calibration set, with the maximum number of LVs set to 16. Subsequently, variable importance in projection (VIP) scores [40] were calculated to evaluate the contribution of each wavelength to the prediction model.

**Table 1:** List of methods used in the three-layer (i.e., 1st, 2nd, and 3rd) preprocessing scheme for spectral images

1st preprocessing	2nd preprocessing	3rd preprocessing
None	None	None
Normalization	Savitzky Golay filter	SNV
Logarithmic transformation	1st derivative	MSC
	2nd derivative	

The available options for the 1st layer were none, corresponding to no preprocessing of the raw data, normalization [36], and logarithmic transformation [38]; the 2nd layer options included none, Savitzky–Golay filtering [35], 1st derivative, and 2nd derivative [39]; and the 3rd layer options were none, standard normal variate (SNV) [37], and multiplicative scatter correction (MSC) [36]. One method was selected from each layer, yielding a total of 36 preprocessing combinations

For all three models, the remaining 20% of the segments were reserved as the test dataset to evaluate predictive performance using the coefficient of determination (*R*²), root mean square error (RMSE), and mean absolute error (MAE), as described below:

1 $$\:{R}^{2}=\:1\:-\:\frac{\sum\:_{i=1}^{n}({y}_{i}-{\widehat{y}}_{i}){\:}^{2}}{\sum\:_{i=1}^{n}{({y}_{i}-{\stackrel{-}{y}}_{i})}^{2}}$$

2 $$\:\mathrm{R}\mathrm{M}\mathrm{S}\mathrm{E}=\:\sqrt{\frac{\sum\:_{i=1}^{n}{({\widehat{y}}_{i}-{y}_{i})}^{2}}{n}}$$

3 $$\:\mathrm{M}\mathrm{A}\mathrm{E}=\:\frac{1}{n}\sum\:_{i=1}^{n}|\widehat{{y}_{i}}-{y}_{i}|$$

Here, $\:{y}_{i}$ is the observed root dry weight of the *i*-th segment, $\:{\widehat{y}}_{i}$ is the predicted root dry weight of the *i*-th segment, $\:\stackrel{\prime }{y}$ is the mean of observed root dry weight across all segments, and *n* represents the total number of segments in the test dataset. The model with the highest accuracy and statistical superiority was then selected to evaluate its applicability to an independent dataset and to visualize the daily accumulation of root biomass.

### Model applicability

#### Data acquisition for model applicability

To evaluate the applicability of the developed model with the highest accuracy under natural root development conditions, an independent experiment was conducted in the same greenhouse from November 2, 2024, to December 22, 2024, using the same spinach cultivar and hydroponic system. For accuracy validation, 104 plants were planted in staggered batches at three-day intervals, from November 2 to November 26, and the root systems of all plants were imaged at final harvest on December 22. In addition, four plants transplanted on November 2 were imaged daily from transplantation through harvest to provide a proof-of-concept demonstration of continuous root biomass monitoring. Following imaging, the roots were separated according to the positions of the planting holes on the hydroponic panel to isolate individual plants. The roots of each plant were then harvested, dried at 80 °C for 72 h, and weighed to determine dry weight. This approach yielded a total of 108 plants, with 104 plants included for accuracy validation and four for visualization. It should be noted that, in this study, two distinct units were used to quantify root biomass: “g/segment” denotes the dry weight of individual root segments (2.5 cm in length) used for model training (Fig. 2a), whereas “g/plant” denotes the total root dry weight of a single plant used for independent validation (Fig. 2b).

#### Model application and root biomass dynamic visualization

The best-performing model was applied to the applicability dataset to estimate root dry weight, and its accuracy was evaluated using *R²* and RMSE (Eqs. 1 and 2). This model was subsequently applied to daily hyperspectral images of the four representative plants to estimate and monitor root biomass dynamics throughout the cultivation period.

### Statistical analyses and software

Image processing and spectral data extraction were performed using MATLAB (Version R2023a; The MathWorks, Inc., Natick, MA, USA), utilizing the Image Processing Toolbox (Version 11.7) and the Hyperspectral Imaging Library (Version 23.1.0). The CNN model was developed in the Python programming language (Version 3.9.13), using the TensorFlow framework (Version 2.10.0) for deep learning implementation and the scikit-learn library (Version 1.2.2) for data preprocessing and performance evaluation. In addition, paired t-tests for statistical comparisons of the three model accuracies (MAE) were performed using the SciPy library (Version 1.10.1). PLSR modeling was conducted in R (Version 4.1.2; R Core Team, 2021). We used the *pls* R package (Version 2.8.0) for model calibration, and the *spectratrait* package (Version 1.1.1) was then used to calculate VIP scores and uncertainty intervals.

## Results

### Relationship between root dry weight and image-derived parameters

**Fig. 5:**
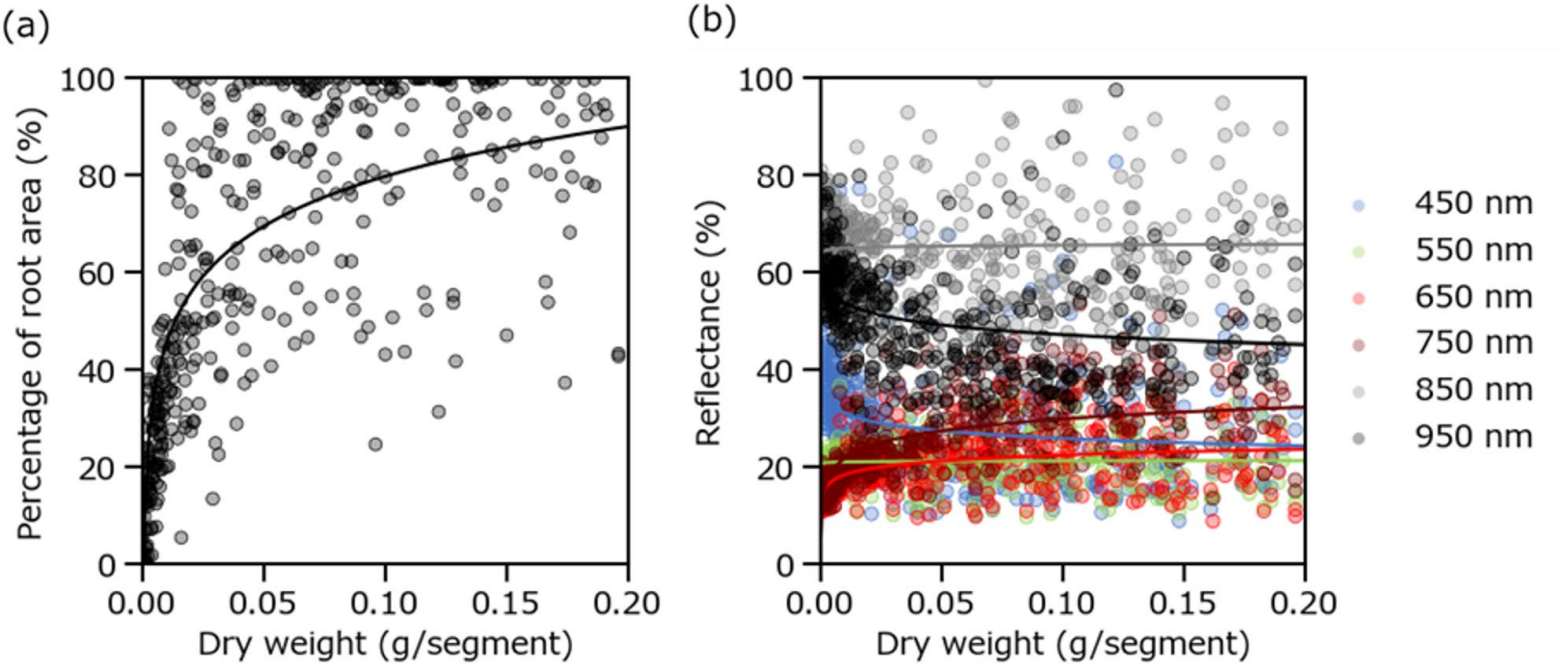
Relationships between the percentage of image area covered by roots in RGB images and root dry weight for each segment (**a**), and between reflectance at different wavelengths (i.e., 450, 550, 650, 750, 850, and 950 nm) in hyperspectral images and root dry weight for each segment (**b**) (*n* = 430)

In RGB images, the percentage of root-covered area exhibited a positive relationship with root dry weight (Fig. 5a, *n* = 430). When dry weight was below 0.050 g/segment, this relationship was approximately linear, indicating that the proportion of root-covered area reliably reflected biomass accumulation at low to moderate levels, corresponding to early growth stages. However, when dry weight exceeded 0.050 g/segment, the distribution of data points became increasingly dispersed, particularly as root-covered area approached 100%. Beyond this point, additional increases in dry weight were no longer captured by the area ratio, indicating saturation of this area-based index.

In hyperspectral images, distinct response patterns were observed at representative wavelengths (i.e., 450, 550, 650, 750, 850, and 950 nm) with increasing root dry weight (Fig. 5b, *n* = 430). Reflectance at 450 nm, corresponding to the blue region, decreased markedly as root dry weight increased, likely because greater root overlap reduced the amount of light reaching and reflecting from the blue hydroponic panel positioned behind the roots. In contrast, reflectance at 750 nm increased with increasing dry weight, whereas reflectance at 950 nm showed a decreasing trend. Overall, wavelengths showing the most pronounced changes associated with root dry weight were concentrated in the near-infrared region (750 nm and 950 nm).

### Comparative model predictive performance

**Fig. 6:**
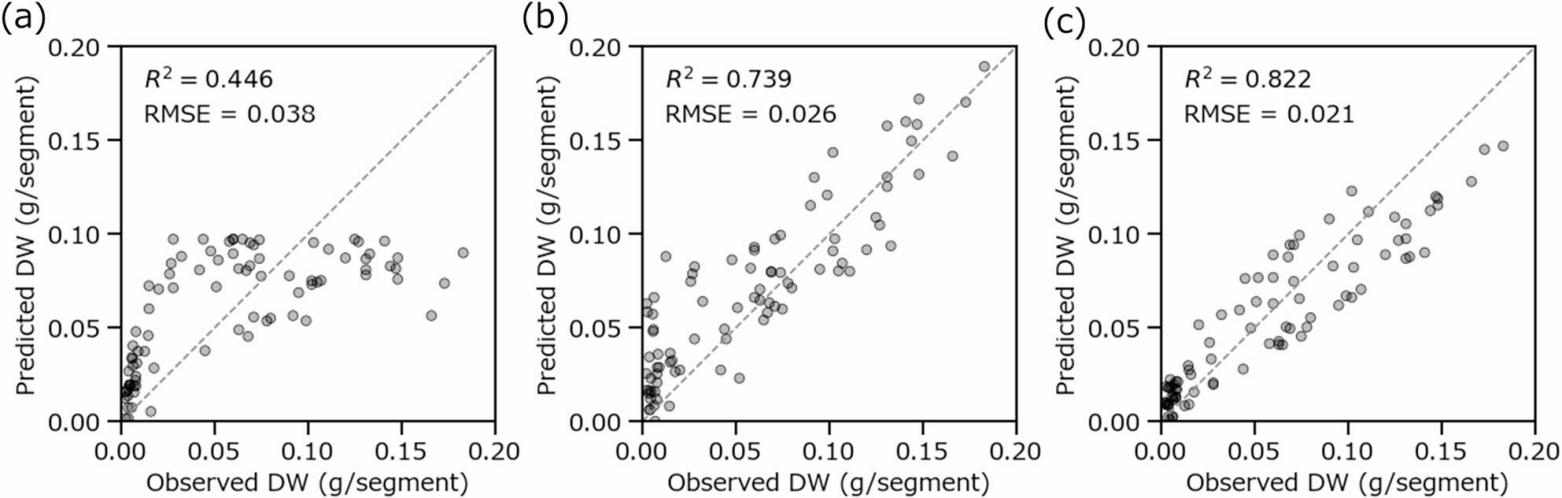
Relationships between observed dry weight and predicted dry weight obtained from the nonlinear model constructed using the percentage of root-covered area (**a**), the CNN model constructed using RGB images (**b**), and the PLSR model constructed using hyperspectral images (**c**) (*n* = 86). CNN, convolutional neural network; PLSR, partial least squares regression

The three models exhibited distinct predictive performance on the test dataset (*n* = 86, representing 20% of the total segments). In the area-based regression model, predicted values showed a positive relationship with observed dry weight; however, overall predictive accuracy remained low (*R*² = 0.446, RMSE = 0.050 g/segment), particularly for segments with higher dry weight (Fig. 6a). This behavior was consistent with the saturation trend observed in Fig. 5a and indicated that the area-based index was unable to capture further increases in biomass once root-covered area reached 100%.

Relative to the area-based approach, the CNN model showed substantially improved overall predictive accuracy (Fig. 6b; *R*² = 0.739, RMSE = 0.023 g/segment). Nevertheless, data points corresponding to dry weights below 0.05 g/segment were widely dispersed and tended to be overestimated, indicating reduced prediction accuracy in this low-biomass range (*R*² = 0.092). These results indicate that, while CNNs can extract morphological and textural features beyond simple area coverage, their predictive performance is limited when root systems are sparse.

In the PLSR model using hyperspectral data (Fig. 6c), predicted dry weights showed the highest agreement with observed values, achieving the largest *R*² (0.822) and a low RMSE (0.019 g/segment) among the three models. This superior performance highlights the advantage of hyperspectral information for capturing subtle spectral variations associated with root physiological and structural properties.

Statistical comparison of model performance using paired t-tests on absolute prediction errors indicated that the CNN and PLSR models significantly outperformed the area-based regression model (*P* < 0.01 and *P* < 0.001, respectively). In addition, the difference in prediction error between the CNN and PLSR models was statistically significant (*P* < 0.01). The PLSR model achieved the lowest MAE (0.018 g/segment), demonstrating superior accuracy in root biomass prediction relative to the CNN model (MAE = 0.020 g/segment).

**Fig. 7:**
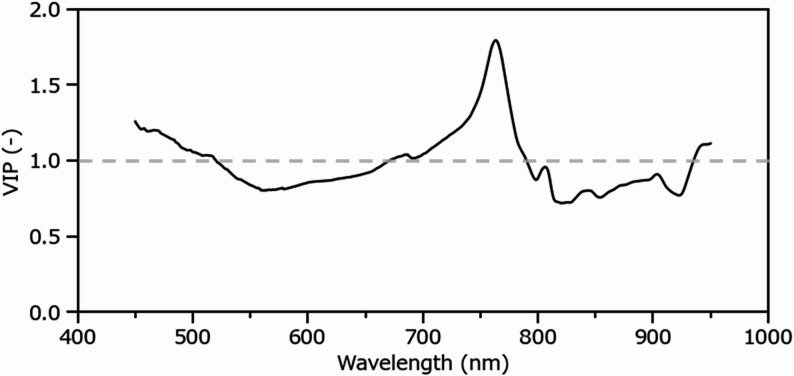
Relationship between VIP scores of predictors in the PLSR model for root dry weight prediction and wavelength. The gray dashed line indicates the threshold of VIP = 1, above which variables are considered to contribute significantly to the model. PLSR, partial least squares regression; VIP, variable importance in projection

The VIP scores derived from the PLSR model are shown in Fig. 7. A VIP score of 1 is commonly used as a threshold to identify variables that contribute significantly to model performance. Notably, VIP scores near 450 nm and 750 nm clearly exceeded this threshold, indicating that spectral information in these regions may be strongly associated with root dry weight.

To visually verify the predictive capability of the PLSR model, Supplementary File 2 presents a representative false-color image of root dry weight generated from a spectral image acquired under linear growth conditions. Root dry weight was highest near the plant base, located at the top of the image, and gradually decreased with increasing distance from the base. This spatial pattern was consistent with natural root growth characteristics, in which roots extend outward from the base, supporting the applicability of this model, although some of the data were included in the training dataset.

### Temporal and spatial prediction of root dry weight under natural root development conditions

**Fig. 8:**
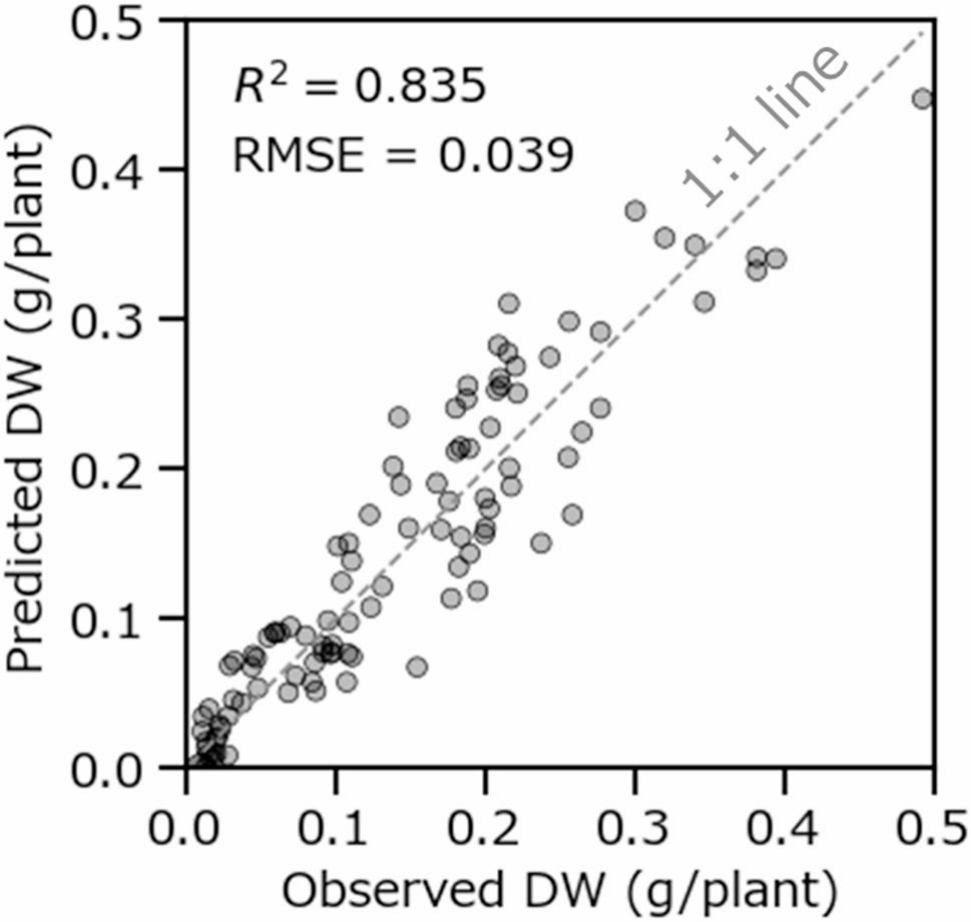
Relationship between observed and PLSR-predicted root dry weight for each plant under natural root development conditions (*n* = 104). *PLSR* partial least squares regression

To evaluate the robustness and applicability of the developed PLSR model, it was further validated using an independent dataset (*n* = 104) collected under natural root development conditions (Fig. 8). The PLSR model exhibited a strong linear correlation between observed and predicted root dry weight, resulting in a high *R*² (0.835) and a low RMSE (0.039 g/plant). These metrics indicate that the model achieved high predictive accuracy even when applied to roots under natural development conditions.

**Fig. 9:**
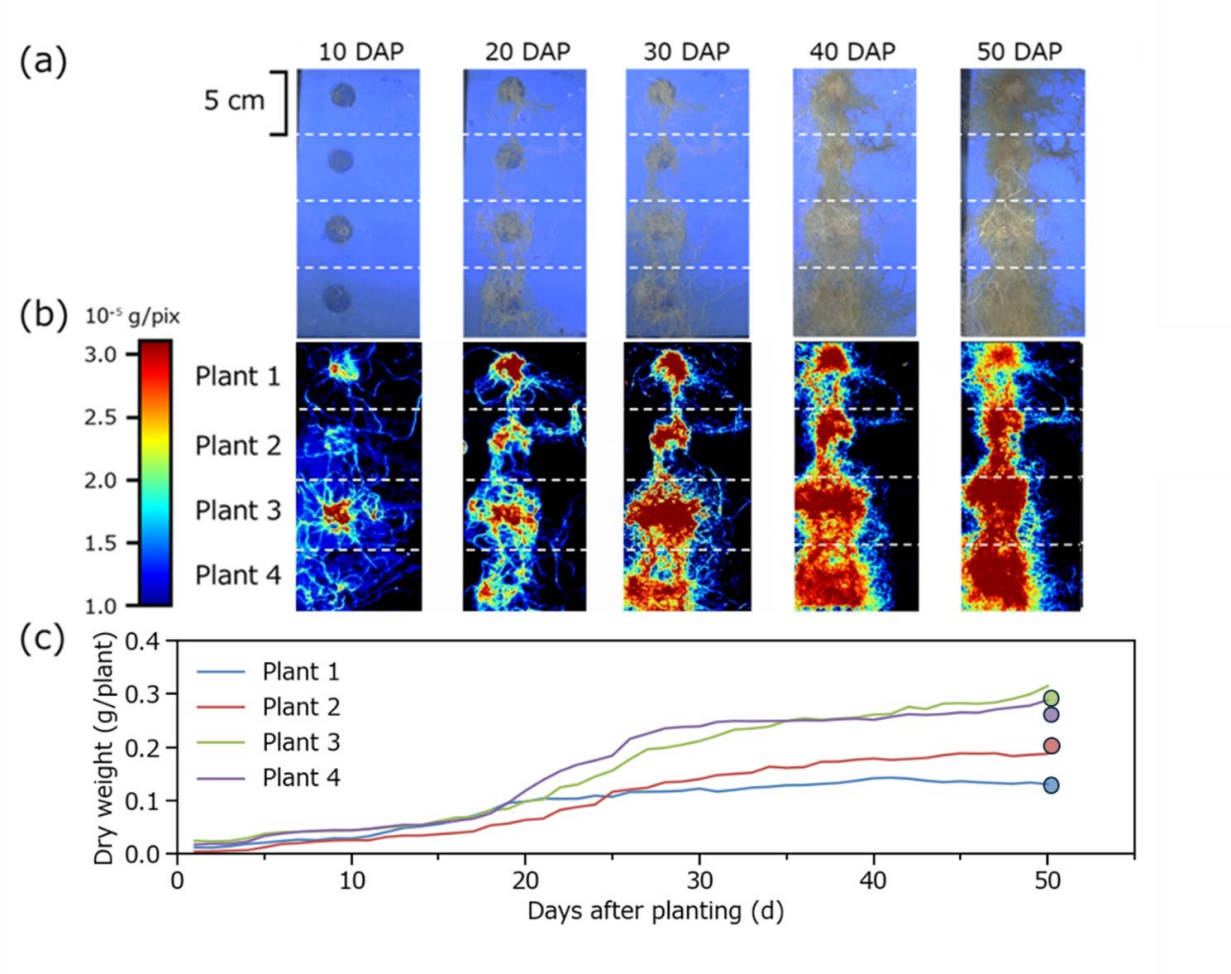
RGB images (**a**) and false-color images (**b**) of root dry weight under natural growth conditions at 10, 20, 30, 40, and 50 DAP, and daily changes in root dry weight of plants 1, 2, 3, and 4 from 0 to 50 DAP (**c**). The data points at 50 DAP represent observed values for each plant at harvest. DAP, days after planting

Next, as a proof-of-concept application, the PLSR model was applied to daily hyperspectral images of four representative plants throughout the 50-day cultivation period to visualize the spatiotemporal dynamics of root biomass accumulation (Fig. 9). Figures 9(a) and (b) show RGB images and corresponding false-color images that represent the root dry weight distribution for the four selected plants. These results show that over time, the roots expanded from the plant’s center toward the periphery. The false-color images revealed a spatial gradient wherein root biomass density gradually decreased from the center to the outer edges, a pattern that was difficult to discern visually in the RGB images. Figure 9(c) illustrates the temporal dynamics of predicted root dry weight for these four plants on a daily basis. Around 20 days after planting (DAP), all plants entered a rapid growth phase, followed by a deceleration in dry weight accumulation after 30 DAP. At 50 DAP, the observed values showed a mean deviation of only 0.017 g, corresponding to a 7.70% relative error, from the predicted values. Overall, this finding provided evidence of robust model performance for estimating root dry weight under natural development conditions.

## Discussion

To the best of our knowledge, this study demonstrates one of the first non-destructive monitoring of entire root system biomass distribution under actual production conditions. The results support our hypothesis that machine learning–based RGB and hyperspectral imaging can be used to predict root dry weight dynamics, with hyperspectral imaging achieving higher accuracy (*R*² = 0.822) because of its richer spectral information. The developed system enabled demonstrative visualization of the spatiotemporal dynamics of root biomass accumulation throughout the cultivation period. Although currently demonstrated on a limited number of individuals, this proof of concept highlights the potential for providing new insights into adaptive root developmental strategies in biomass allocation and spatial distribution in response to environmental variations. In the following sections, these findings are discussed from two perspectives: (1) model performance and spectral interpretation, and (2) practical applications, limitations, and future perspectives.

### Model performance and spectral interpretation

The performance of three models developed under linear growth conditions was first evaluated using the test dataset. The limited capacity of the area-based model (Fig. 6a; *R*² = 0.446) to capture biomass accumulation during later growth stages underscores that simple morphological coverage is insufficient for complex root systems, thereby supporting the necessity of advanced approaches that capture textural or spectral features. The CNN model achieved an *R*² of 0.739 for root dry weight prediction (Fig. 6b), although it was not able to resolve spatial distribution in the same manner as the PLSR model. This level of performance is comparable to that reported in CNN-based biomass estimation studies focusing on above-ground systems. For example, Zhang et al. [19] reported an *R*² of 0.891 for greenhouse lettuce leaf dry weight prediction using a CNN, while Ma et al. [41] achieved an *R*² of 0.808 for the prediction of winter wheat above-ground dry weight using a pre-designed deep CNN. More recent studies have shown that CNN-based approaches can achieve *R*² values ranging from 0.78 to 0.90 when sufficient image resolution and training data are available [19–21, 41]. The *R*² value obtained for the CNN model in this study (0.739) falls within this range. However, prediction accuracy was substantially lower for sparse root segments (*R*² = 0.092; Fig. 5(b), dry weight < 0.05 g/segment). This reduction in accuracy is likely attributable to the low spatial resolution of the input segments (86 × 43 pixels), which was constrained by the integrated RGB sensor of the hyperspectral camera system used for simultaneous acquisition. Although images were upscaled to the model input size (224 × 224 pixels), conventional interpolation cannot recover high-frequency textural information lost during image acquisition [42]. Consequently, limited spatial resolution constrained the extraction of fine-grained textural features from sparse roots, thereby reducing the model’s predictive performance in the low-biomass range. Although this study prioritized simultaneous data acquisition with an integrated system, the use of a standalone high-resolution RGB camera would likely improve feature extraction and predictive accuracy for sparse roots, while increasing equipment costs.

In contrast, the PLSR model using hyperspectral data achieved an *R*² of 0.822 with an RMSE of 0.019 g/segment (Fig. 6c), indicating superior and more stable performance across the full biomass range. This accuracy is consistent with or exceeds that reported in many PLSR-based studies of above-ground biomass prediction. For instance, Song et al. [28] reported *R*² values of 0.85 and 0.88 for shoot and root dry weight prediction, respectively, in *Arabidopsis* using near-infrared hyperspectral imaging with wavelength optimization, while studies on wheat dry weight [43] achieved *R*² values of 0.78 using PLSR. In addition, studies on leaf mass per area have shown that PLSR can achieve *R*² values of 0.89 using full-range leaf reflectance spectra across environments ranging from the Arctic to the tropics [44]. Notably, the PLSR model in this study maintained consistent accuracy despite the same resolution constraints that limited CNN performance, indicating that spectral information provides more robust indicators of biomass than morphological features alone. This stability across different root densities highlights a key advantage of hyperspectral imaging, namely its ability to capture physiological and structural properties through spectral signatures rather than relying solely on visual morphological features that may be obscured due to sparse distribution or overlap.

Despite relying on less spectral information than hyperspectral imaging, the CNN model achieved slightly lower yet comparable accuracy. This modest performance gap suggests that the CNN model effectively compensates for the lack of reduced spectral resolution by processing raw pixel-level RGB images that preserve spatial information, including root morphology, shape, and area distribution. In contrast, the PLSR model uses spatially averaged spectral reflectance, aggregating pixel-level spectral characteristics across multiple wavelengths. By capturing spectral signatures associated with internal tissue properties rather than morphological features alone, the PLSR model maintained stable accuracy across the full biomass range, overcoming the saturation effects observed in the area-based model and the sparse-root limitations of the CNN model. To clarify the mechanistic basis of this advantage, we further examined the spectral interpretation underlying the superior performance of the PLSR model. A subsequent VIP analysis (Fig. 7) indicated that model performance was driven primarily by two spectral mechanisms, background contrast in the blue region around 450 nm and tissue scattering in the near-infrared region around 750 nm [45]. Specifically, the 450 nm signal corresponds to light that penetrates the root system, reflects from the blue background panel, and passes back through the roots to the sensor. As root overlap increases, transmittance decreases with increasing optical path length, leading to a pronounced reduction in observed blue reflectance. Although this mechanism produces a strong correlation with biomass, it is inherently limited. Once root overlap becomes sufficiently dense to block the return of light reflected from the background panel, blue reflectance reaches a minimum threshold (Fig. 5b), restricting accurate estimation of further biomass accumulation and explaining the slight underestimation observed for high-biomass segments (Fig. 6c). It should be noted that the prominence of 450 nm is partly attributable to the use of a blue background panel, which was selected to maximize spectral contrast with root tissue and to facilitate root–background segmentation [29]. If alternative background colors were used, different wavelength regions would be expected to exhibit high VIP scores, corresponding to spectral characteristics at which the new background and root tissue show maximum contrast. However, the fundamental principle that wavelengths capturing background–root interaction contribute to biomass estimation would remain applicable. In contrast, the signal at 750 nm depends primarily on internal tissue properties rather than background information. This wavelength lies within the red-edge spectral region (680–750 nm). While this region is typically associated with pigment transitions in foliage, for non-photosynthetic root tissues, the reflectance at 750 nm is primarily governed by internal light scattering at cellular structures such as cell wall composition and intercellular air spaces [46]. Since near-infrared reflectance (700–1000 nm) is influenced by cell wall–air interfaces, it remains sensitive to biomass accumulation driven by tissue thickening and lignification [45–47]. This capacity to detect physiological changes, even in dense root systems, reinforces the critical advantage of hyperspectral imaging over RGB-based methods.

To further validate this advantage of PLSR, the model was evaluated under natural root development conditions. Despite differences in root growth patterns between linear and natural cultivation conditions, the PLSR model achieved comparable estimation accuracy under both conditions (Figs. 6c and 8), indicating that the data captured in the linear cultivation dataset sufficiently encompass the characteristics of naturally developed roots. This finding also suggests that the CNN model developed from the linear cultivation dataset exhibits similar predictive performance for naturally developed roots. However, regarding spatial visualization, the CNN model can only predict the total dry weight at the segment-size level (as defined in the training data ), whereas the PLSR model enables pixel-level prediction of dry weight, thereby allowing high-resolution spatial visualization of biomass distribution throughout the root system, as demonstrated in Fig. 9.

### Practical applications, limitations, and future perspectives

Non-destructive monitoring of root biomass offers significant advantages for data-driven crop production, including smart and precision agriculture. Unlike destructive sampling methods, which provide information only at discrete time points, the system described here enables continuous and non-destructive assessments of root growth dynamics without disturbing plant development. As demonstrated by the model’s robust performance under natural root development conditions (Fig. 8) and the spatiotemporal visualizations of root biomass (Fig. 9), this capability reveals biomass patterns that are difficult to capture using traditional methods. Overall, this finding provides actionable insights for environmental control and crop management. Furthermore, implementation is achievable via simple modification of existing NFT systems by installing transparent baseplates. However, a significant trade-off exists between sensor cost and accuracy: hyperspectral cameras are suitable for research and high-throughput phenotyping of root growth traits. Conversely, multispectral cameras represent an intermediate option between hyperspectral and RGB approaches, offering a more economical alternative for use at agricultural sites while retaining key spectral information [48]. Moreover, this system also facilitates fundamental eco-physiological research by enabling continuous tracking of root-shoot allocation dynamics, root stress responses, and root growth patterns within individual plants. Crucially, non-destructive monitoring approaches can compensate for the specific limitations of established methodologies by circumventing issues caused by disruption and inter-individual variability when using destructive sampling, while simultaneously overcoming the partial visibility and scalability issues typical of existing non-destructive techniques.

Despite promising results, we acknowledge several limitations regarding this study. First, the developed models were calibrated for this specific experimental setup using a blue background panel. Consequently, changes in background color may affect predictive performance, particularly for the PLSR model, by shifting the spectral bands that contribute most substantially to the predictions. Future studies should examine model transferability across different background conditions or establish adaptive calibration protocols to ensure robustness in diverse experimental environments. Second, the CNN and PLSR models were validated exclusively on spinach and require further experimentation to achieve general applicability to crops exhibiting different root morphologies resulting from distinct cultivation conditions, cultivars, or species. Crops exhibiting substantially different root morphologies, such as the fibrous root systems of green onions and Chinese chives, would likely require model retraining or recalibration because architectural differences in roots may modify spectral signatures. Similarly, alternative cultivation systems (e.g., deep water culture, and aeroponics) may introduce variation in background reflectance and root spatial distribution that could influence model performance. Third, in the comparative study design, both RGB and hyperspectral images were acquired using the same integrated camera system, enabling simultaneous data collection under identical conditions; however, this approach also implies that RGB imaging was not evaluated at its full potential owing to the limited spatial resolution of the integrated RGB sensor. The use of dedicated high-resolution RGB cameras or advanced hyperspectral systems with greater spatial or spectral resolution may produce different comparative outcomes.

Future research should focus on enhancing the robustness and generalizability of model predictions. First, expanding the training dataset to encompass a wider range of biomass values, cultivation conditions, cultivars, and species could improve model performance across diverse scenarios. Second, integrating RGB and hyperspectral data through advanced fusion strategies, including ensemble modeling [49, 50] or multi-modal deep learning [51, 52], may effectively integrate the strengths of RGB and hyperspectral imaging.

## Conclusions

In conclusion, this study presents a novel approach for non-destructive, continuous monitoring of complete root system biomass in hydroponic production systems. The ability to visualize spatiotemporal dynamics of root development represents a clear methodological advancement relative to conventional destructive sampling approaches. Although hyperspectral imaging combined with PLSR achieved the highest predictive accuracy, the comparative evaluation of three distinct approaches provides practical guidance for researchers and growers in selecting appropriate methods based on specific objectives, available resources, and required accuracy. As sensor technologies continue to mature and associated costs decline, integration of non-destructive phenotyping systems into commercial production facilities is expected to become increasingly feasible, thereby supporting more sustainable and efficient crop production through improved understanding and management of root system development.

## Electronic Supplementary Material

Below is the link to the electronic supplementary material.

Supplementary Material 1.

## Data Availability

The datasets generated and analyzed during the model construction of the current study are available in the Zenodo repository: [https://zenodo.org/records/18072801?preview=1&token=eyJhbGciOiJIUzUxMiJ9.eyJpZCI6ImFiYTkzMzY2LTIzZjktNDlkMy1iZTBjLTk3M2E5YTUyOTFmZCIsImRhdGEiOnt9LCJyYW5kb20iOiIzNGE1ZjMxNDZhYjhiYjlhZWRiOWFjNzBkNzcwY2I3NyJ9.uR4HfosoSaVWhtSblMOS1v9bJFA5MvHwXvcW9uoNbcTWRDU4RNxZpVHjXTC3ulBM1JTlBbeHp_4T5EcILawxdg].The dataset includes: - Raw hyperspectral images and data- RGB images - Root dry weight measurements

## Abbreviations

CNN: Convolutional neural network
DAP: Days after planting
EC: Electrical conductivity
HSI: Hyperspectral imaging
LV: Latent variable
MAE: Mean absolute error
MSC: Multiplicative scatter correction
NFT: Nutrient film technique
PLSR: Partial least squares regression
RMSE: Root mean square error
SNV: Standard normal variate
VIP: Variable importance in projection
