# Supplementary material for: Non-destructive monitoring of root biomass in hydroponically grown leafy vegetables: comparison between machine learning-based RGB and hyperspectral imaging
Source: Plant Methods. 2026 Mar 10;22:38. doi: 10.1186/s13007-026-01515-8 (PMC13088466; doi:10.1186/s13007-026-01515-8)
Supplement: Supplementary file 1 — Supplementary Material 1. [file 13007_2026_1515_MOESM1_ESM.docx]

**Supplementary Material**

**Supplementary File 1**

| Coefficient of determination for the prediction of PLSR models constructed using datasets preprocessed by the 36 combinations listed in Table 1. PLSR, partial least squares regression; SNV, standard normal variate; MSC, multiplicative scatter correction. |
| --- |
| 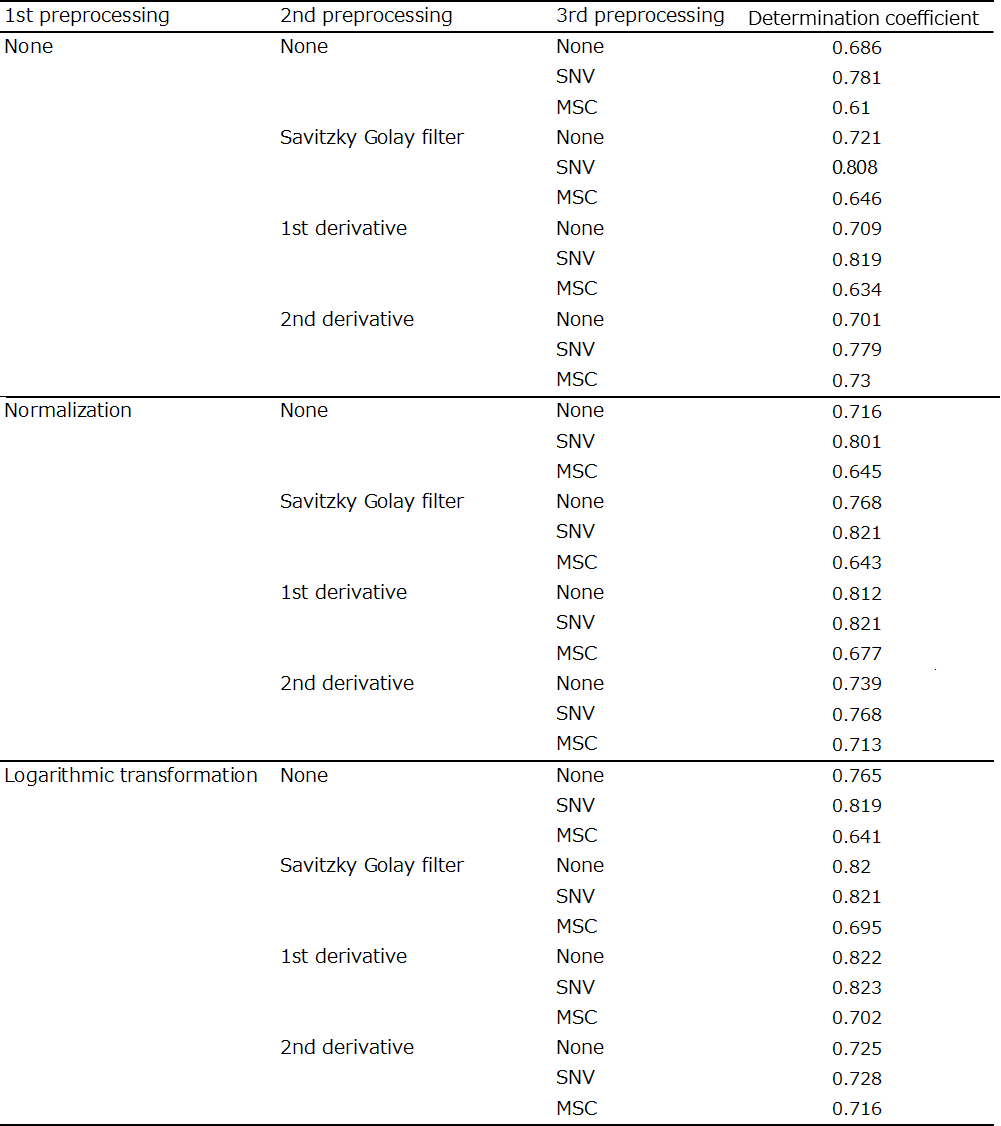 |

**Supplementary File 2**

| 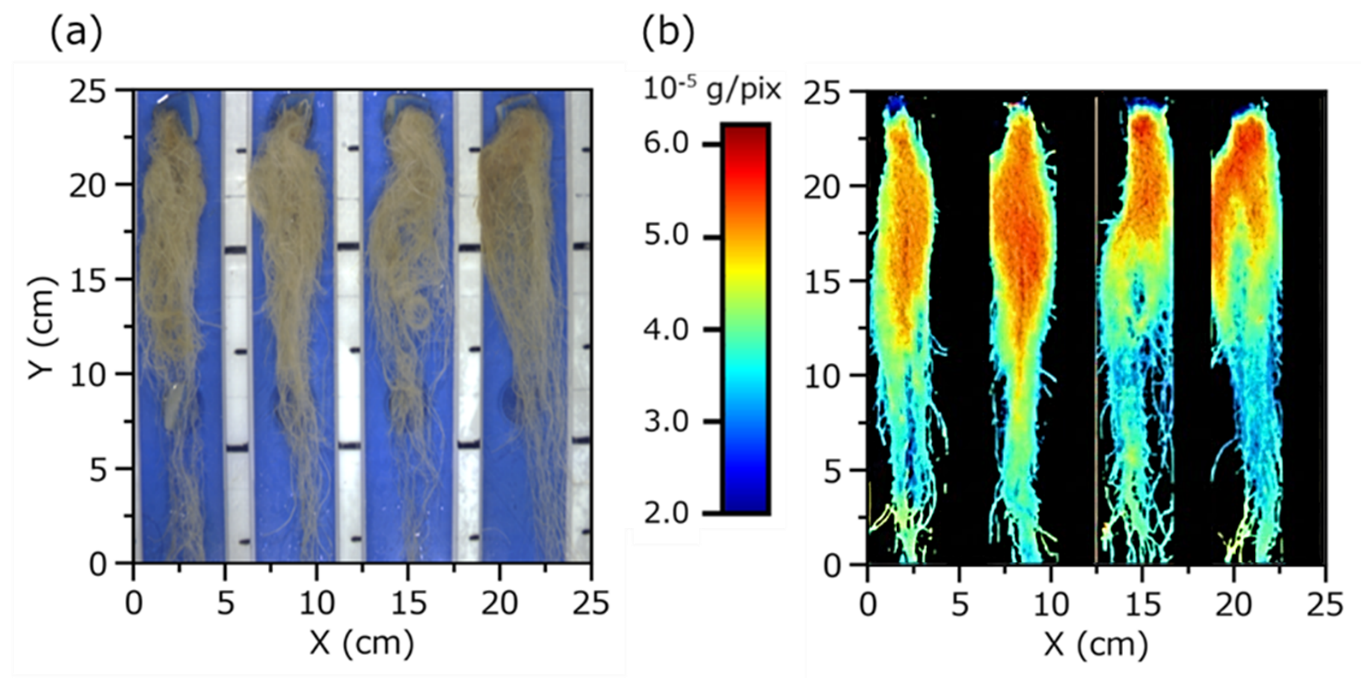 |
| --- |
| RGB image of roots grown under a linear growth condition (a) and the corresponding false-color image representing root dry weight (b). |
